# Interleukin-1β Level Is Increased in Vitreous of Patients with Neovascular Age-Related Macular Degeneration (nAMD) and Polypoidal Choroidal Vasculopathy (PCV)

**DOI:** 10.1371/journal.pone.0125150

**Published:** 2015-05-15

**Authors:** Min Zhao, Yujing Bai, Wankun Xie, Xuan Shi, Fangting Li, Fei Yang, Yaoyao Sun, Lvzhen Huang, Xiaoxin Li

**Affiliations:** 1 Department of Ophthalmology, Peking University People’s Hospital, Beijing, China; 2 Key Laboratory of Vision Loss and Restoration, Ministry of Education, Beijing, China; 3 Beijing Key Laboratory of Diagnosis and Therapy of Retinal and Choroid Diseases, Beijing, China; Univeristy of Miami, UNITED STATES

## Abstract

**Purpose:**

To examine the expression of pro-interleukin-1β (pro-IL-1β) and interleukin-1β (IL-1β) in the vitreous body of patients with neovascular age-related macular degeneration(nAMD), polypoidal choroidal vasculopathy (PCV), proliferative diabetic retinopathy (PDR), retinal vein occlusion (RVO) or Eales’ disease to further elucidate the role of IL-1β and inflammation in the pathogenesis of neovascular retinal disease.

**Design:**

Prospective clinical laboratory investigation study.

**Methods:**

All patients enrolled had vitreous hemorrhage due to nAMD, PCV, PDR, RVO or Eales’ disease that required vitrectomy. Patients were excluded for any history of active intraocular inflammation, or other ophthalmic surgery besides vitrectomy. Control samples were obtained from patients with idiopathic macular epiretinal membrane. A total of fifty vitreous samples were collected from patient during vitrectomy. Pro-IL-1β and IL-1β expression were measured by enzyme-linked immunosorbent assay (ELISA). Results were analyzed statistically using nonparametric tests.

**Results:**

Expression of pro-IL-1β protein was increased by 2.83-fold and 9.19-fold in PCV and nAMD vitreous samples relative to control, respectively. Expression of IL-β protein was increased by 10-fold and 4.83-fold in PCV and nAMD vitreous samples relative to control, respectively.

**Conclusions:**

Our results demonstrate that expression of pro-IL-1β and IL-1β proteins is higher in PCV and nAMD. The roles of pro-IL-1β and IL-1β as inflammatory mediators in the development of PCV and nAMD may be associated with photoreceptor degeneration and neovascularization which necessitates further study.

## Introduction

As the leading cause of irreversible blindness in the elderly, age-related macular degeneration (AMD) is associated with many risk factors, including genetic and environmental factors such as oxidative stress and inflammation [1–3]. Polypoidal choroidal vasculopathy (PCV) is described as a special form of choroidal vasculopathy distinct from neovascular AMD (nAMD) with choroidal neovascularization (CNV). Idiopathic PCV is distinguishable from other choroidal neovascular disorders by its several unique clinical features and angiographic findings. However, there remains some controversy over whether or not PCV represents a subtype of AMD.

Inflammation has been shown to be associated with a variety of retinal diseases including AMD, PCV and proliferative diabetic retinopathy (PDR) [4]. Both the innate and adaptive immune systems are activated in these diseases. Various cytokines associated with protection and innate immunity are recruited in inflammatory eye diseases, including pattern recognition receptors (PRRs)[5].

Interleukin-1β (IL-1β) is a key pro-inflammatory cytokine which can initiate innate immune processes associated with inflammation, infection, and autoimmunity [6, 7]. IL-1β is secreted in an inactive pro-interleukin-1β (pro-IL-1β) form and requires proteolytic cleavage by caspase-1 to be released in its active form [8]. Caspase-1, also known as IL-1 converting enzyme (ICE), is a cysteine protease that specifically cleaves the 31kD pro-IL-1β precursor to produce the mature, 17kD active IL-1β [8, 9]. The caspase-1 activation platform, referred to as the inflammasome, links the sensing of pathogen and danger signals to pro-IL-1β activation[10]. The NLRP3 inflammasome has been previously reported to be involved in AMD [11–13]. Previous studies showed that caspase-1 activity increased in galactosemic mice and diabetic patients. C-reactive protein (CRP), a nonspecific marker of acute and chronic inflammation, was shown to be elevated in both PCV and nAMD [14, 15]. Furthermore, evidence of local inflammatory responses has been found both in macular drusen deposits [16] and in arterioles near drusen-like deposits [17–19].

IL-1β binds to the interleukin-1 receptor (IL-1R), which activates the myeloid differentiation factor 88 (MyD88) adapter protein to initiate a downstream signaling pathway [20]. IL-1β is associated with chronic inflammatory disease and its expression is maintained at a relatively constant level [14, 21, 22]. Although IL-1β was shown to be the major product of inflammasome cascade activation, levels of pro-IL-1β and IL-1β in the vitreous body from eyes with nAMD, PCV and other retinal vascular disease have not been previously studied.The present study attempted to address this issue by by simultaneously analyzing the expression of pro-IL-1β and IL-1β in the vitreous obtained during vitrectomy using an enzyme-linked immunosorbent assay (ELISA). Expression in vitreous from diseased eyes was compared to that in vitreous from eyes with idiopathic macular epiretinal membrane only.

## Methods

### Patients

Our prospective study was performed with the approval of the Ethical Committee of Peking University People’s Hospital and was conducted in accordance with the Declaration of Helsinki.

All participants gave written informed consent and were subsequently enrolled between April 2012 and January 2013. This prospective series included vitreous samples from fifty patients with vitreous hemorrhage due to nAMD with CNV, PCV, PDR, RVO or Eales’ disease who had received vitreous aspiration during vitrectomy. Ten patients with nAMD, ten with PCV, eight with PDR, eight with Eales’ disease and eight with RVO were enrolled. Six samples from patients with idiopathic macular epiretinal membrane were used as the control group (Table 1).

**Table 1:** The concentration of IL-1β protein level.

IL-1β	Mean concentration(pg/ml)	SEM	N	P value
control	0.41	0.19	6	
PDR	0.67	0.17	8	-
nAMD	1.98	0.49	10	*
PCV	4.20	0.5	10	**
Eales’	1.55	0.43	8	*
RVO	1.60	0.09	8	*

*<0.05 compared with control group

**P<0.01 compared with control group

- no significantly change compared with control group

All patients received a standard ophthalmic examination by a retinal specialist (Dr. Xuan Shi and Dr. Xiaoxin Li). The diagnoses of nAMD and PCV were made according to the International Classification System for AMD and the classification system for PCV [23–26]. Each patient also received fluorescein angiography (FA), optic coherence tomography (OCT), and indocyanine green angiogram (ICGA) testing.

All patients underwent vitrectomy, performed by Dr. Xuan Shi and Dr. Xiaoxin Li at the Peking University People’s Hospital, Beijing, China, after approval by the hospital’s institutional review board.

Patients were enrolled that had vitreous hemorrhage due to nAMD, PCV, PDR, RVO or Eales’ disease that required vitrectomy, and that had not had any other ophthalmic surgery previously. Prior to enrollment, all patients gave written informed consent to have vitreous aspiration sampling performed during vitrectomy. Any patients who were receiving intravitreal injection treatment during the period of enrollment, who had active intraocular inflammation, recent cerebral vascular accident or myocardial infarction, or had other systemic diseases which precluded vitrectomy were excluded from the study. Patients with idiopathic macular epiretinal membrane and underwent vitrectomy were selected as the control group.

### Sample Collection

The sampling procedure was undergone during the 3-port lens-sparing vitrectomy with manual suction by sterile syringes. Approximately 100μl undiluted vitrectomy samples were obtained from the midvitreous of patients. The intraocular irrigating solution (Alcon) was not opened until the procedure completed.

The undiluted vitreous samples were aspirated by sterile syringes and centrifuged at 3000 rpm for 10 minutes at 4°C to remove cells and debris, then immediately transferred to store at -80°C until the time of assay. The technician and doctor involved in the study were masked to all the samples.

### ELISA

Pro-IL-1β and IL-1β expression of all the samples were measured by enzyme-linked immunosorbent assay (ELISA) using a kit for human anti- pro-IL-1β (DLBP00; R&D Systems) and a kit for human anti- IL-1β (QLB00B; R&D Systems). Each assay was performed according to the manufacturer’s instruction. The optical density was determined at 450 nm using an absorption spectrophotometer. The optical density mean values were reading for three times for quantitative analysis.

A standard curve for this assay was built with recombinant human pro-IL-1β and IL-1β (R&D Systems).

### Statistical Analysis

Results were analyzed statistically using nonparametric tests because of the skewed distribution (Kruskal-Wallis variance analysis and Bonferroni corrected Mann–Whitney *U* test) and were expressed as the median and range. A *P* value less than 0.05 was considered statistically significant. Statistical analysis was performed using SPSS software version 11.5 (SPSS, Inc., Chicago, IL).

## Results

### The concentrations of IL-1β Levels in the Vitreous Samples

The concentrations of IL-1β in nAMD, PCV, RVO, PDR, Eales' disease and idiopathic macular epiretinal membrane patients’ vitreous samples were measured by ELISAs. Our results showed that the concentration of IL-1β was 0.41±0.19 pg/ml (mean±SEM) in control group. However, the concentration of IL-1β was 4.20±0.5 pg/ml in PCV vitreous samples and 1.98±0.49 pg/ml in nAMD vitreous samples. At the same time, the concentration of IL-1β was 1.60±0.09 pg/ml in RVO vitreous samples and 1.55±0.43 pg/ml in Eales’ disease vitreous samples. The concentration of IL-1β was 0.67±0.17 pg/ml in PDR vitreous samples (Table 1).

### The concentrations of pro-IL-1β Levels in the Vitreous Samples

The concentrations of pro-IL-1β in nAMD, PCV, RVO, PDR, Eales' disease and idiopathic macular epiretinal membrane patients’ vitreous samples were measured by ELISAs. Our results showed that the concentration of pro-IL-1β was 21.63±5.15 pg/ml (mean±SEM) in control group. However, the concentration of IL-1β was 198.91±73.79pg/ml in nAMD vitreous samples and 119.19±73.86 pg/ml in RVO vitreous samples. At the same time, the concentration of pro-IL-1β was 61.23±5 pg/ml in PCV vitreous samples and 72.03±17.71 pg/ml in Eales’ disease vitreous samples. The concentration of IL-1β was 36.09±14.03pg/ml in PDR vitreous samples (Table 2).

**Table 2:** The concentration of Pro-IL-1β protein level.

Pro-IL-1β	Mean Concentration(pg/ml)	SEM	N	P value
control	21.63	5.15	6	
PDR	36.09	14.03	8	-
nAMD	198.91	73.79	10	*
PCV	61.23	5.0	10	*
Eales’	72.03	17.71	8	*
RVO	119.19	73.86	8	-

*<0.05 compared with control group

**P<0.01 compared with control group

- no significantly change compared with control group

### IL-1β Levels were markedly elevated in the PCV, nAMD, Eales’ disease and RVO Vitreous Samples

**Fig 1:**
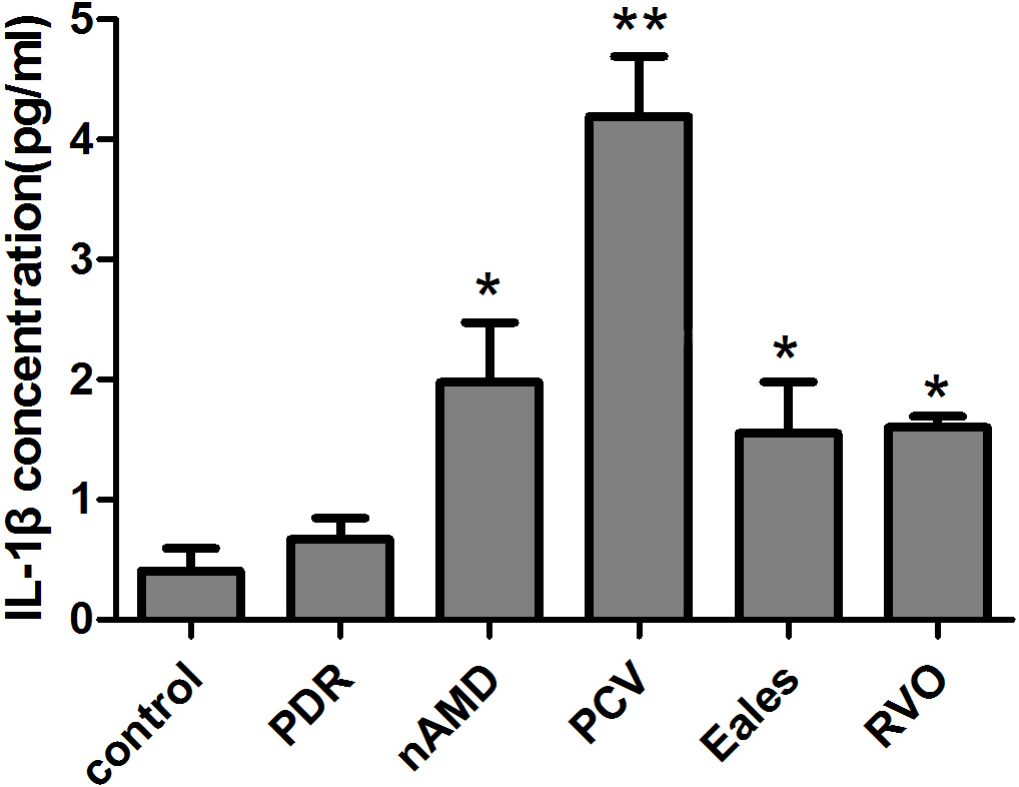
The concentration of IL-1β protein in vitreous samples. The concentrations of IL-1β in nAMD, PCV, RVO, PDR, Eales' disease and idiopathic macular epiretinal membrane patients vitreous samples were measured by ELISAs. The concentration of IL-1β was 4.20±0.5 pg/ml in PCV vitreous samples and 1.98±0.49 pg/ml in nAMD vitreous samples and 1.55±0.43 pg/ml in Eales’ disease vitreous samples and 1.60±0.09 pg/ml in RVO vitreous samples, which were markedly elevated when compared with control group (0.41±0.19 pg/ml; p<0.05) (mean±SEM).

The concentrations of IL-1β levels in the vitreous samples were listed in Table 1. The concentration of IL-1β in PCV, nAMD, Eales’ disease and RVO vitreous samples were significantly elevated when compared with control group (Table 1, Fig 1). A 10-fold and 4.83-fold increase of IL-1β protein expression was detected in PCV (P<0.01) and nAMD (P<0.05) vitreous body respectively compared to control group.

### Pro-IL-1β Levels were markedly elevated in the PCV, nAMD and Eales’ disease Vitreous Samples

**Fig 2:**
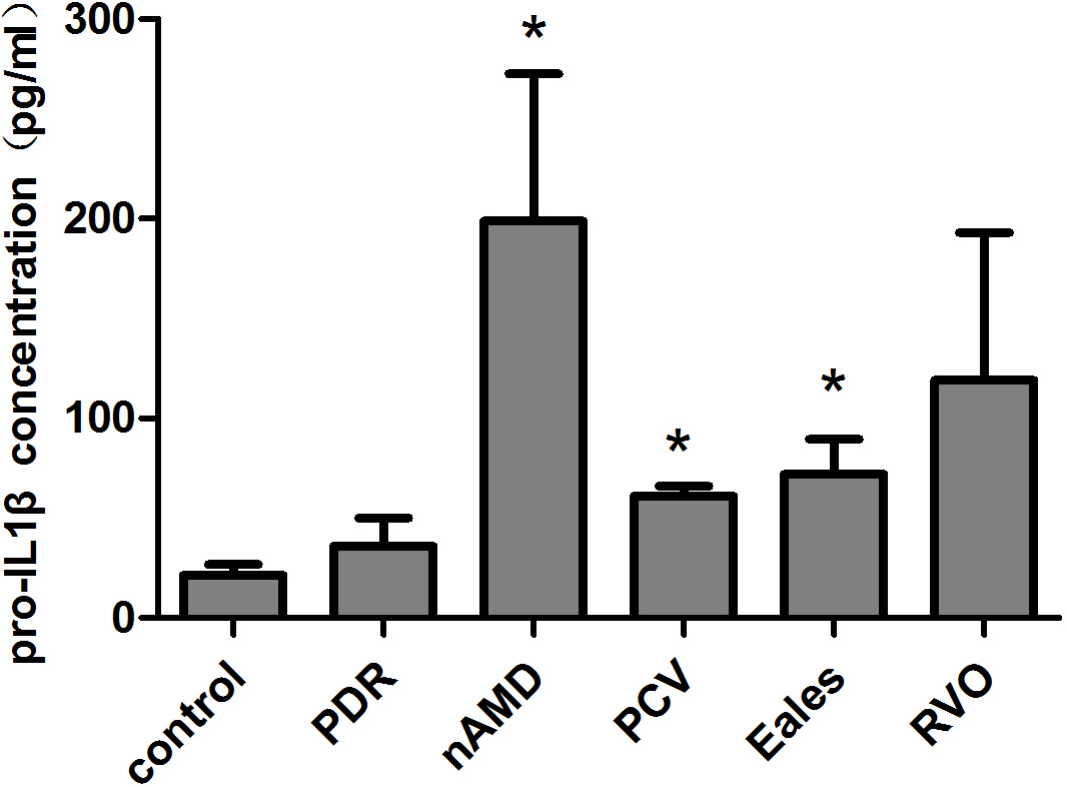
The concentration of Pro-IL-1β protein in vitreous samples. The concentrations of pro-IL-1β in nAMD, PCV, RVO, PDR, Eales' disease and idiopathic macular epiretinal membrane patients vitreous samples were measured by ELISAs. The concentration of pro-IL-1β was 198.91±73.79 pg/ml in nAMD vitreous samples and 61.23±5 pg/ml in PCV vitreous samples and 72.03±17.71 pg/ml in Eales’ disease vitreous samples, which were markedly elevated when compared with control group (21.63±5.15 pg/ml; p<0.05) (mean±SEM).

The concentrations of pro-IL-1β levels in the vitreous samples were listed in Table 2. The concentration of pro-IL-1β in nAMD, PCV and Eales’ disease vitreous samples were significantly elevated when compared with control group (Table 2, Fig 2). A 2.83-fold and 9.19-fold increase of pro-IL-1β protein expression was detected in PCV (P<0.05) and nAMD (P<0.05) vitreous body respectively compared to control group.

## Discussion

There are more and more evidence for local inflammatory responses in the pathophysiology of AMD, PDR, RVO, Eales’ disease and PCV [16, 27, 28]. IL-1β, a key pro-inflammatory cytokine, can promote inflammation cascades and play an important role in these diseases [14, 21, 22]. Inflammasome links the sensing of pathogen and danger signals to pro-IL-1β activation. NLRP3 inflammasome is also reported to be involved in AMD which plays a role on detecting components of bacteria [11].

IL-1β is the major factor to active inflammasome cascades. However, there is few data on inflammatory cytokines expression in AMD and PCV vitreous samples. We therefore analyzed the protein level of pro-IL-1β and IL-1β in vitreous samples from patients with nAMD, PCV and other retinal vascular diseases. Our result showed that pro-IL-1β and IL-1β levels were markedly elevated in the PCV, nAMD vitreous samples compared to control group. This indicated that the IL-1β related inflammatory pathway may be associated with nAMD and PCV. However, we were unable to distinguish nAMD and PCV from vitreous samples on the basis of the expression of IL-1β or pro-IL-1β proteins, though PCV samples contained higher levels of IL-1β while nAMD samples contained higher levels of pro-IL-1β. The reason remains unclear as the number of subjects enrolled in our present study was limited. We assumed that IL-1β might be involved in both diseases although nAMD and PCV have different etiology, pathology and mechanisms.

Previous studies showed that the level of IL-1β increased in retinas of diabetic rats. The IL-1β expression was also reported to be elevated in the vitreous of PDR and Eales’ disease patients[27, 29–32]. Another study showed controversial result that the IL-1β was detected in only 10.3% vitreous samples from PDR patients at very low levels[33]. Our result showed that pro-IL-1β and IL-1β levels were elevated in the Eales’ disease vitreous samples compared to control group. These results indicated that IL-1β maybe involved in the Eales’ disease which was consistent with previous studies[27].

However, interestingly, from our results, pro-IL-1β and IL-1β levels were not markedly elevated in the PDR vitreous samples which differed from most of previous studies[27, 32, 34]. Currently, it is believed that the vascular barrier breakdown in the diabetic retinopathy and inflammation may play a role in the diabetic retinopathy[35]. It is a very long process that non proliferative diabetic retinopathy develops into proliferative diabetic retinopathy. Thus, the intraocular cytokines levels should be a dynamic process too. One study showed that the IL-1β level in DR patients’ aqueous humor differed with different DR severities and increased as the DR progressed[34]. We assumed that the level of pro-IL-1β and IL-1β levels were not markedly elevated in the PDR vitreous samples in our study maybe related to the DR severities. Patients enrolled in our study were diagnosed with vitreous hemorrhage due to PDR which was the milder stage of PDR. The other reason maybe because that the number of patients diagnosed with PDR in our study was limited.

nAMD, PCV, RVO, PDR and Eales’ disease have different etiology, pathology and demonstrate individual features. The symptoms and signs of these diseases are with great difference. Immunoinflammatory mechanism may be involved in those diseases but may not have a similar inflammation cytokine expression pattern in the vitreous. The capillary circulation is disturbed, the capillary endothelial cells is injured in DR. Then, minute NPAs is formed and become more and more extensive as the disease progresses, causing neovascularization and the formation of fibrovascular membrane in the interface between the retina and vitreous body[36]. Conversely, there is a pigmental epithelial (RPE) cell dysfunction and the choroidal vessels are dilated in AMD. Then new vessels sprout from the choroidal vessels, penetrate the Bruch’s membrane and grow into the subretinal space. Indeed, the RPE cell is one of the most important systems that regulate the immune response in the eye. Thus, the pathway between RPE damage and ocular inflammation is likely a two way street whereby RPE damage potentiates inflammation, which further worsens RPE degeneration [1]. Such a relatively RPE cell dysfunction may be one reason that the inflammatory reaction in nAMD is more severe than in DR.

RPE cells were the key immune cells in the macula immune defence. The dysfunction of RPE cells could trigger the inflammatory response, which could further trigger the photoreceptors degeneration and neovascularization development [11, 37–39]. This might be one of the mechanisms of PCV and nAMD development.

IL-1β was involved in the abnormal angiogenesis process. However, the reason that IL-1β plays different role in the development of PCV and nAMD remains unknown. One possibility was that IL-1β was upregulated in both PCV and nAMD conditions but with different level and time window which may further trigger different cell signaling pathways to activate different abnormal angiogenesis process. The increased expression of IL-1β in PCV and nAMD patients raises a possibility that the inflammasome could be involved in these diseases. Previous studies concluded controversial conclusion on the role of inflammasome in AMD [40]. Some concluded that inflammasome was harmful and the other concluded that it is beneficial [13, 41]. Inflammasome may play a dual role in AMD. The role of inflammasome and this hypothesis needs to be verified in our further studies.

In addition, an analysis of the IL-1β levels in the serum have been studied and listed in the supplemental data (S1 Text and S1 Fig) to demonstrate whether the higher concentrations of IL-1β in the vitreous of nAMD and PCV patients are due to the increased concentration in the serum. Our results in the supplemental data showed that there was a significant decrease in the concentration of IL-1β in PCV (P<0.05) and nAMD(P<0.01) serum samples compared with control group. Thus, we assumed that the decrease in the concentration of IL-1β in the blood of nAMD and PCV patients may not interference the result on the increase in the concentration of IL-1β in the vitreous samples of nAMD and PCV patients.

Taken together, our results suggested that IL-1β related inflammatory mechanism may be associated with nAMD and PCV. This association could be a result of the activation of inflammation, which further stimulates RPE cells to trigger the photoreceptors degeneration and neovascularization. The different role of IL-1β in the development of PCV and nAMD needs to be verified in further studies.

## Supporting Information

S1 Fig The concentrations of IL-1β Levels in the serum Samples. The concentrations of IL-1β in nAMD, PCV and idiopathic macular epiretinal membrane patients’ serum samples were measured by ELISAs. The concentration of IL-1β was 2.28±0.17 pg/ml (mean±SEM) in control group. The concentration of IL-1β was 0.53±0.14 pg/ml in PCV serum samples and 0.47±0.12 pg/ml in nAMD serum samples. There was a significant decrease in the concentration of IL-1β in PCV (P<0.05) and nAMD (P<0.01) serum samples compared with control group.
(TIF)

S1 Text The concentrations of IL-1β Levels in the serum Samples of nAMD and PCV patients. The concentrations of IL-1β in nAMD, PCV and idiopathic macular epiretinal membrane patients’ serum samples were measured by ELISAs to determine the intereference of blood on the result. The results showed that the decrease in the concentration of IL-1β in the blood of nAMD and PCV patients may not interference the result on the increase in the concentration of IL-1β in the vitreous samples of nAMD and PCV patients.
(DOC)
