# Supplementary figures and images for: Interleukin-1β Level Is Increased in Vitreous of Patients with Neovascular Age-Related Macular Degeneration (nAMD) and Polypoidal Choroidal Vasculopathy (PCV)
Source: PLoS One. 2015 May 15;10(5):e0125150. doi: 10.1371/journal.pone.0125150 (PMC4433218; doi:10.1371/journal.pone.0125150)

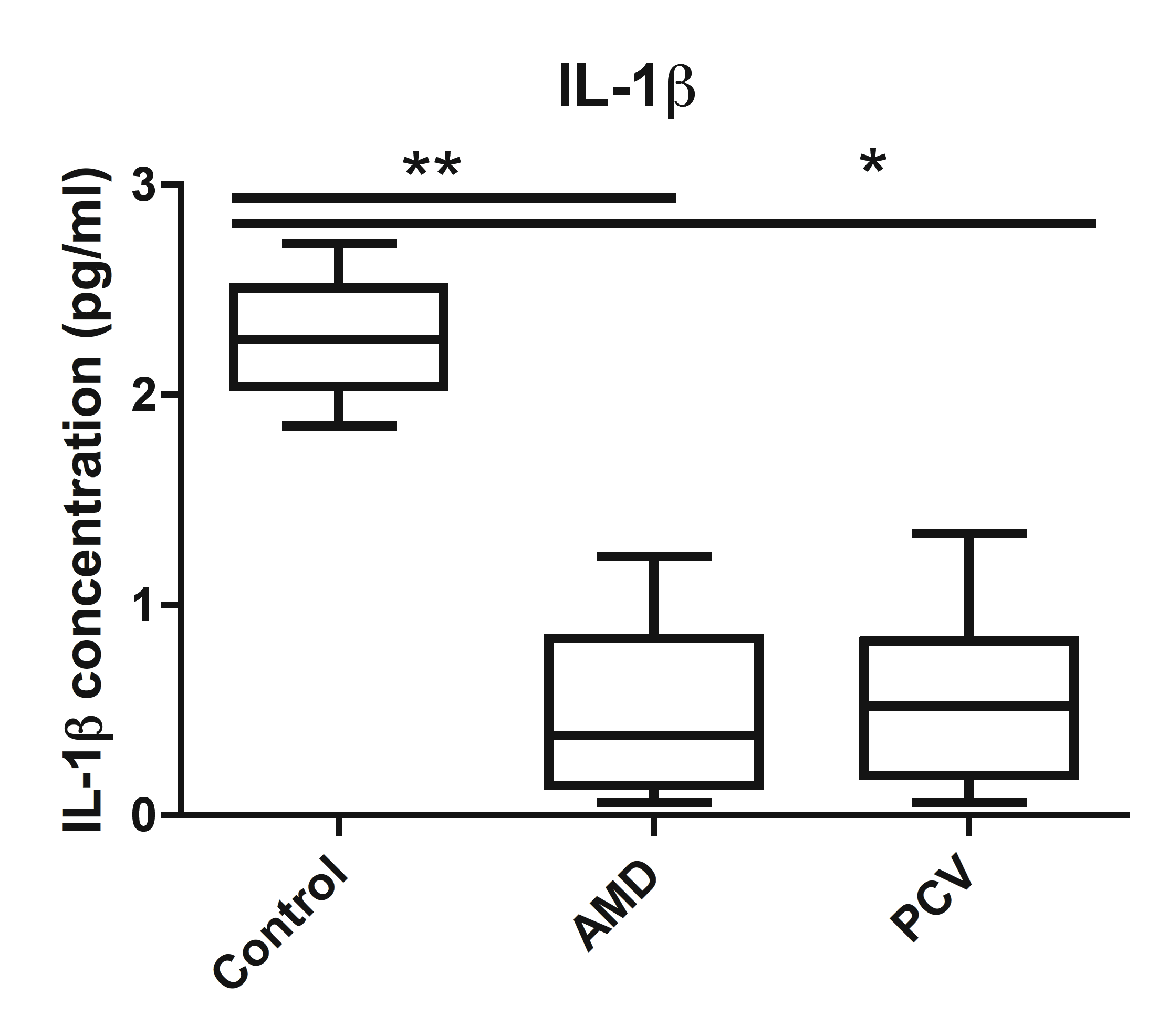

Supplement: S1 Fig — The concentrations of IL-1β in nAMD, PCV and idiopathic macular epiretinal membrane patients’ serum samples were measured by ELISAs. The concentration of IL-1β was 2.28±0.17 pg/ml (mean±SEM) in control group. The concentration of IL-1β was 0.53±0.14 pg/ml in PCV serum samples and 0.47±0.12 pg/ml in nAMD serum samples. There was a significant decrease in the concentration of IL-1β in PCV (P<0.05) and nAMD (P<0.01) serum samples compared with control group. (TIF) [file pone.0125150.s001.tif]
