# Supplementary material for: Interleukin-1β Level Is Increased in Vitreous of Patients with Neovascular Age-Related Macular Degeneration (nAMD) and Polypoidal Choroidal Vasculopathy (PCV)
Source: PLoS One. 2015 May 15;10(5):e0125150. doi: 10.1371/journal.pone.0125150 (PMC4433218; doi:10.1371/journal.pone.0125150)
Supplement: S1 Text — The concentrations of IL-1β in nAMD, PCV and idiopathic macular epiretinal membrane patients’ serum samples were measured by ELISAs to determine the intereference of blood on the result. The results showed that the decrease in the concentration of IL-1β in the blood of nAMD and PCV patients may not interference the result on the increase in the concentration of IL-1β in the vitreous samples of nAMD and PCV patients. (DOC) [file pone.0125150.s002.doc]

**Supplemental data**

**Method**

**Patients**

Our prospective study was performed with the approval of the Ethical Committee of Peking University People’s Hospital and was conducted in accordance with the Declaration of Helsinki. All participants gave written informed consent and were subsequently enrolled between April 2012 and January 2013.

This part of study included vitreous samples and blood samples from 20 patients with vitreous hemorrhage due to nAMD with CNV or PCV who had received vitreous aspiration during vitrectomy. Prior to vitrectomy, 5ml blood sample was taken for each patient. Ten patients with nAMD, ten with PCV were enrolled. Six samples from patients with idiopathic macular epiretinal membrane were used as the control group. Blood sample was also obtained in control group.

All patients received a standard ophthalmic examination as previously described in this manuscript. Patients were enrolled that had vitreous hemorrhage due to nAMD or PCV that required vitrectomy, and that had not had any other ophthalmic surgery as previously described in this manuscript. Patients with idiopathic macular epiretinal membrane and underwent vitrectomy were selected as the control group.

**Sample Collection**

Prior to vitrectomy, 5ml blood sample was taken for each patient using collection tubes and leave for 1 hour at room temperature. Then the serum samples were separated form blood sample by centrifugation at 3000 rpm for 10 minutes at 4°C at room temperature. All the serum samples were stored in sterile tubes and stored at -80°C until the time of assay. The technician and doctor involved in the study were masked to all the samples.

**ELISA**

The concentrations of IL-1β in nAMD, PCV and idiopathic macular epiretinal membrane patients’ serum samples were measured by ELISAs (QLB00B; R&D Systems) to determine the intereference of blood on the result. The ELISA assay is the same as described previously in this manuscript.

**Statistical Analysis**

Results were analyzed statistically using the same method as described previously in this manuscript.

**Result**

**The concentrations of IL-1β Levels in the serum Samples**

The concentrations of IL-1β in nAMD, PCV and idiopathic macular epiretinal membrane patients’ serum samples were measured by ELISAs. Our results showed that the concentration of IL-1β was 2.28±0.17 pg/ml (mean±SEM) in control group. However, the concentration of IL-1β was 0.53±0.14 pg/ml in PCV serum samples and 0.47±0.12 pg/ml in nAMD serum samples. There was a significant decrease in the concentration of IL-1β in PCV (P<0.05) and nAMD (P<0.01) serum samples compared with control group (S1 Fig).
